# Clinical outcomes of bariatric surgery in patients with obesity and idiopathic intracranial hypertension

**DOI:** 10.1007/s00464-024-11254-3

**Published:** 2024-10-04

**Authors:** Mélissa V. Wills, Mohammad Hesam Alavi, Essa M. Aleassa, Chao Tu, Rickesha Wilson, Ricard Corcelles, Toms Augustin, Kalman P. Bencsath, Walter Cha, Jesse Gutnick, Samuel Szomstein, Raul Rosenthal, Matthew Kroh, Xiaoxi Feng, Ali Aminian

**Affiliations:** 1 Department of General Surgery, Cleveland Clinic, Bariatric and Metabolic Institute, Cleveland, OH USA; 2 Department of General Surgery, Digestive Diseases Institute, Cleveland Clinic Abu-Dhabi, Abu Dhabi, United Arab Emirates; 3 Department of Quantitative Health Sciences, Cleveland Clinic, Lerner Research Institute, Cleveland, OH USA; 4 Department of General Surgery, Cleveland Clinic Florida, Bariatric and Metabolic Institute, Weston, FL USA

**Keywords:** Idiopathic intracranial hypertension, Bariatric surgery, Gastric bypass, Sleeve gastrectomy, Obesity, Headache, Intracranial pressure, Papilledema

## Abstract

**Introduction:**

Obesity is a major risk factor for idiopathic intracranial hypertension (IIH). Effective therapeutics for preventing disease progression and alleviating symptoms are limited. This study aims to examine the effects of bariatric surgery on clinical outcomes of IIH.

**Methods:**

We retrospectively collected data from the medical record of 97 patients with obesity and an existing diagnosis of IIH who underwent primary bariatric surgery at the Cleveland Clinic health system in the USA between 2005 and 2023. Pre- and postoperative data on presence of symptoms and clinical markers of IIH (headaches, visual field defects, papilledema, visual symptoms), intracranial pressure, and usage of IIH medications were compared.

**Results:**

A total of 97 patients (98% female, median age 46.7 years, median BMI 48.3 kg/m^2^) with IIH who underwent bariatric surgery including Roux-en-Y gastric bypass (*n* = 66, 68%), sleeve gastrectomy (*n* = 27, 27.8%), and gastric banding (*n* = 4, 4.1%) were analyzed. In a median follow-up time of 3.0 years, the median total weight loss was 24% (interquartile range, 13–33%). There was a significant improvement in headache, papilledema, visual field deficits, and visual symptoms after bariatric surgery. The mean lumbar opening pressure before and after bariatric surgery was 34.8 ± 8.2 cm CSF and 24.2 ± 7.6 cm CSF, respectively, with a mean reduction of 10.7 cm CSF (95% confidence interval, 4.7 to 16.6), *p* = 0.003. The dosage of acetazolamide and topiramate, as well as the number of medications taken for IIH, decreased significantly after bariatric surgery (*p* < 0.001).

**Conclusion:**

For patients who have obesity, bariatric surgery is a viable treatment modality for alleviation or improvement of symptoms of IIH.

Idiopathic intracranial hypertension (IIH) is a chronic neurological disease that manifests with severe headaches and papilledema, in the absence of a secondary cause of increased intracranial pressure. It can result in persistent and debilitating symptoms, including visual disturbances, nausea, vomiting, tinnitus, hearing loss, spontaneous cerebrospinal rhinorrhea or leak, and progression to blindness if left untreated [1]. The pathogenesis is thought to be related to cerebral flow dynamics, and though not entirely understood, is closely linked to obesity. Seventy to 90% of patients with IIH are estimated to have obesity [2]. The incidence of IIH, which at one time was a rare disease, has mirrored obesity trends and has therefore become a significant medical burden in the USA over the past several decades [3].

The current available treatments for IIH, such as carbonic anhydrase inhibitors, anticonvulsants, cerebrospinal fluid (CSF) diversion procedures, and emergent decompressive procedures, are intended to alleviate its symptoms [4, 5]. However, weight loss is a disease-modifying therapy. Prior studies have demonstrated that bariatric surgery can have favorable outcomes in intracranial pressure (ICP) reduction and symptomatic improvements [6]. Smaller observational studies and systematic analyses have demonstrated that bariatric surgery is associated with quality-of-life improvements [7]. However, the relationship between ICP, weight loss, and symptoms is not clear and less is known about whether bariatric surgery influences the need for adjuvant treatments, such as pharmacotherapy, especially in the long-term setting.

To address the current knowledge gap, the present study sought to evaluate the effectiveness of bariatric surgery in treating IIH symptoms. The goal of the analysis was to specifically compare the use of medications and treatment adjuncts before and after bariatric surgery as a proxy for improvement of disease.

## Methods

### Study population and data collection

This study was a retrospective cohort analysis. The charts of 127 patients with IIH who received bariatric surgery at the Cleveland Clinic health system (in Ohio and Florida hospitals) between 2005 and 2023 were evaluated for data collection. Records were identified using the International Classification of Diseases, Tenth Revision (ICD-10) code for idiopathic intracranial hypertension (G93.2) and ICD-9-CM Diagnosis Code 348.2, as well as Current Procedural Terminology (CPT) codes for laparoscopic sleeve gastrectomy (43775), Roux-en-Y gastric bypass (43847), and gastric band placement (43774).

Following electronic identification, each chart underwent manual review to ascertain diagnosis of IIH. Patients with symptomatic manifestations (e.g., papilledema or visual field defects), patients undergoing pharmacological management with IIH medications (e.g., topiramate, acetazolamide), or invasive interventions (including therapeutic lumbar puncture, ventriculoperitoneal shunt placement, ventriculoatrial shunt placement, or venous stenting) were included. Furthermore, patients with diagnostic lumbar puncture opening pressures were selected into the study cohort.

Male and female patients of all ages were included. The surgery date and type (Roux-en-Y gastric bypass, sleeve gastrectomy, or adjustable gastric banding) were recorded. Length of follow-up was defined using the time between the bariatric surgery and the last outpatient postoperative visit to either a bariatric surgeon or neurosurgeon. Preoperative and postoperative weight (kg) and BMI (kg/m^2^) were defined as the weight measured at the last preoperative bariatric clinic visit and at last follow-up, respectively.

Data regarding the type and number of preoperative and postoperative invasive procedures for IIH were recorded. Ventriculoperitoneal shunts, lumboperitoneal shunts, ventriculoatrial shunts, venous stents, or craniotomies done at any time before or after the bariatric surgery were included. To examine the ICP, preoperative and postoperative lumbar puncture (LP) opening pressures were collected if available.

Preoperative presence and absence, as well as postoperative changes, of visual field deficits, papilledema, and visual symptoms were collected via chart review. Presence of visual field deficits was defined via Humphrey visual testing. Presence of papilledema was documented by ophthalmoscopy or visual exam, and presence of visual symptoms was defined by subjective patient report of visual symptoms. These were each collected at the last preoperative visit and at the last follow-up.

### Outcomes

The primary outcome of the study was the change in the number of IIH medications that patients took from before to after bariatric surgery. Data regarding the number, type, and dosage of medications for symptom control of IIH were collected. These included the two first-line IIH medications, topiramate and acetazolamide, as well anti-epileptics in the absence of pre-existing history of epilepsy, pain medications (oxycodone), NSAIDs (ibuprofen), and diuretics (furosemide) if noted in the chart to be used specifically for treatment of IIH.

Other outcomes included changes in IIH symptoms, ICP, body weight, and BMI after bariatric surgery.

### Statistical analysis

Categorical data were presented as number (%) and compared using the McNemar test. Continuous data were presented as median (interquartile range [IQR]) and the Wilcoxon sign rank test was used to test the paired difference in these outcomes. For the paired comparison of ICP (mean ± standard deviation) before and after bariatric surgery, a paired *t* test was used.

## Results

There were 97 patients with IIH that underwent bariatric surgery. Most patients were female (*N* = 95, 97.9%) and most underwent Roux-en-Y gastric bypass (*N* = 66, 68%). The median age at the time of surgery was 37.7 (IQR, 30.4–45.1 years). The median follow-up was 3.0 years (IQR 1.1–6.8 years).

The median preoperative weight and BMI were 130 kg (IQR, 116.1–142.3 kg) and 48.3 kg/m^2^ (IQR, 43.5–52.0 kg/m²), respectively. The median postoperative weight and BMI were 97.8 kg (IQR, 81.0–116.5 kg) and 36.7 kg/m^2^ (IQR, 30.9–43.9 kg/m²), respectively (Table 1). In the last follow-up time, the median total weight loss was 24% (IQR, 13%-33%).

**Table 1:** Demographic and clinical characteristics of 97 patients with idiopathic intracranial hypertension who underwent bariatric surgery

Characteristic	Count (%) or median (IQR)
Age (years)	37.7 (30.4–45.1)
Female	95 (97.9%)
Median preoperative weight (kg)	130 (116.1–142.3)
Median preoperative BMI (kg/m^2^)	48.3 (43.5–52.0)
Median weight loss, kg	31.6 (17.8–44.0)
Roux-en-Y gastric bypass (*N* = 66)	36.7 (24.3–49.2)
Sleeve gastrectomy (*N* = 23)	19.9 (8.6–32.9)
Adjustable gastric band (*N* = 4)	13.9 (6.7–22.9)
Median follow-up, years	3.0 (1.1–6.8)

Categorical data are presented as number (%) and continuous data are presented as median (IQR)

*BMI* body mass index

There was a significant improvement or resolution of headache (*p* < 0.001), visual field deficits (*p* < 0.001), papilledema (*p* < 0.001), and visual symptoms (*p* = 0.007), postoperatively.

**Fig. 1:**
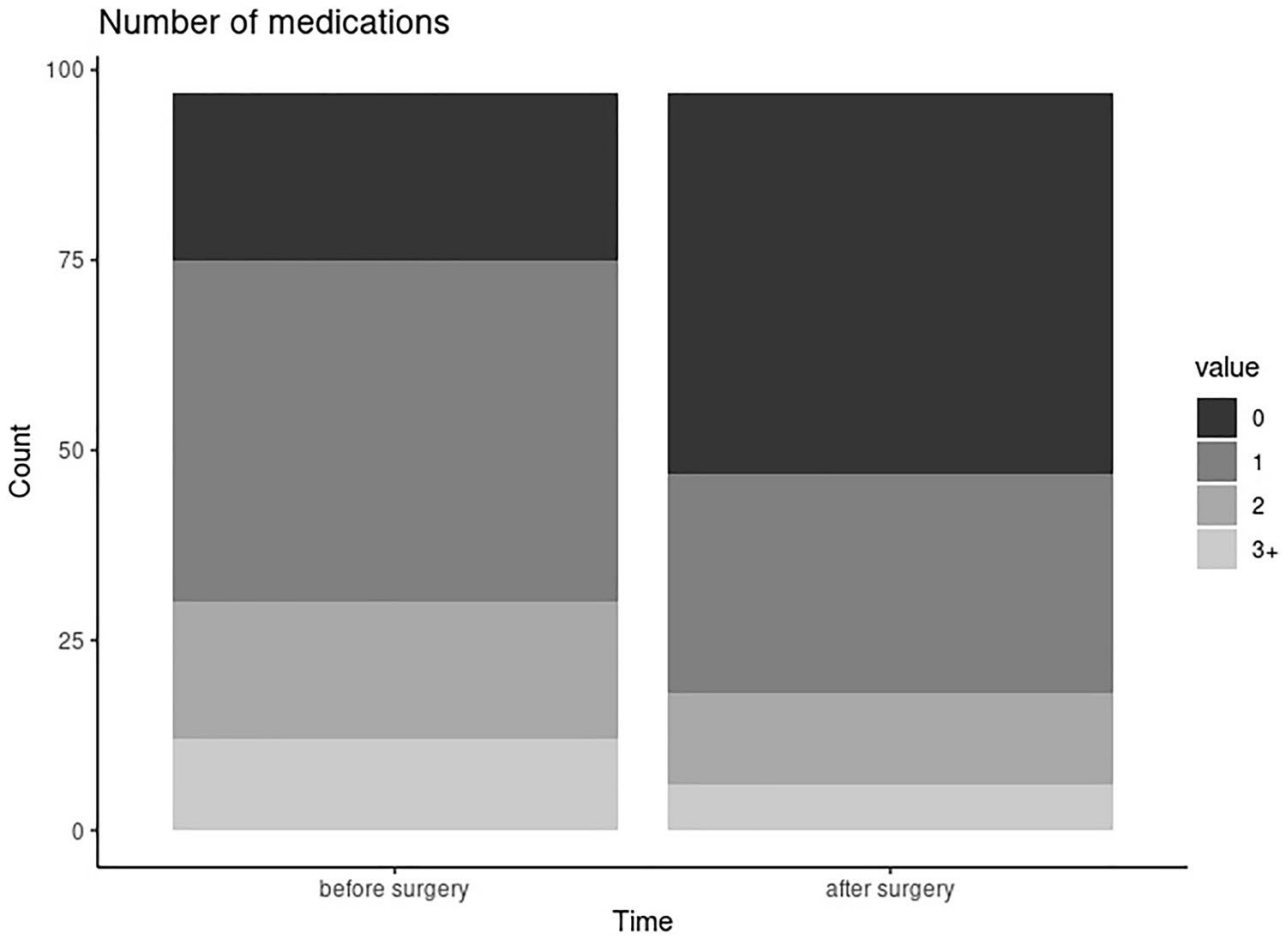
Distribution of number of medications for idiopathic intracranial hypertension taken before and after bariatric surgery

There was a significant decrease in the number of IIH medications taken postoperatively compared to preoperatively (*p* < 0.001) (Fig. 1), and a significant decrease in the dosage of both topiramate and acetazolamide (*p* < 0.001 for both) (Table 2). Patients underwent more invasive procedures (ventriculoperitoneal, lumboperitoneal, and ventriculoatrial shunts; craniectomy; venous stents) preoperatively (*N* = 27) than postoperatively (*N* = 17), but the difference in the number of procedures done preoperatively and postoperatively did not reach statistical significance (*p* = 0.090).

**Table 2:** Outcomes of symptom burden, invasive procedures, and medication use before and after bariatric surgery

	Number of patients with symptoms, invasive treatments, and medications		
	Preoperative	Postoperative	*p*-value
Symptom			
Headache	83 (85.6%)	58 (59.8%)	< 0.001
Papilledema	47 (48.5%)	10 (10.3%)	< 0.001
Visual symptoms	48 (49.5%)	24 (24.7%)	0.007
Visual field deficits	23 (23.7%)	12 (12.4%)	< 0.001
Invasive treatments			
Total	27 (27.8%)	17 (17.5%)	0.090
VP shunt	14 (14.3%)	9 (9.3%)	
LP shunt	6 (6.2%)	4 (4.1%)	
Venous stent	3 (3.1%)	1 (1.0%)	
Optic nerve fenestration	3 (3.1%)	1 (1.0%)	
VA shunt	1 (1.0%)	1 (1.0%)	
Craniotomy	0 (0.0%)	1 (1.0%)	
Medications			
Number of patients taking medications	75 (77.3%)	47 (48.5%)	< 0.001
Number of medications			
0	22 (22.7%)	50 (51.5%)	
1	45 (46.4%)	29 (29.9%)	
2	18 (18.6%)	12 (12.4%)	
≥ 3	12 (12.4%)	6 (6.2%)	
Acetazolamide^a^	49 (51.0%)	16 (16.7%)	
Decreased dose	–	39 (40.6%)	< 0.001
No change	–	52 (54.2%)	
Increased dose	–	5 (5.2%)	
Topiramate^a^	40 (41.7%)	19 (19.8%)	
Decreased dose	–	29 (30.2%)	< 0.001
No change	–	57 (59.3%)	
Increased dose	–	10 (10.4%)	

Categorical data are presented as number (%)

*VP* ventriculoperitoneal, *LP* lumboperitoneal, *VA* ventriculoatrial

^a^One patient on Acetazolamide and Topiramate did not have dosage data available and therefore, the denominator was 96

**Fig. 2:**
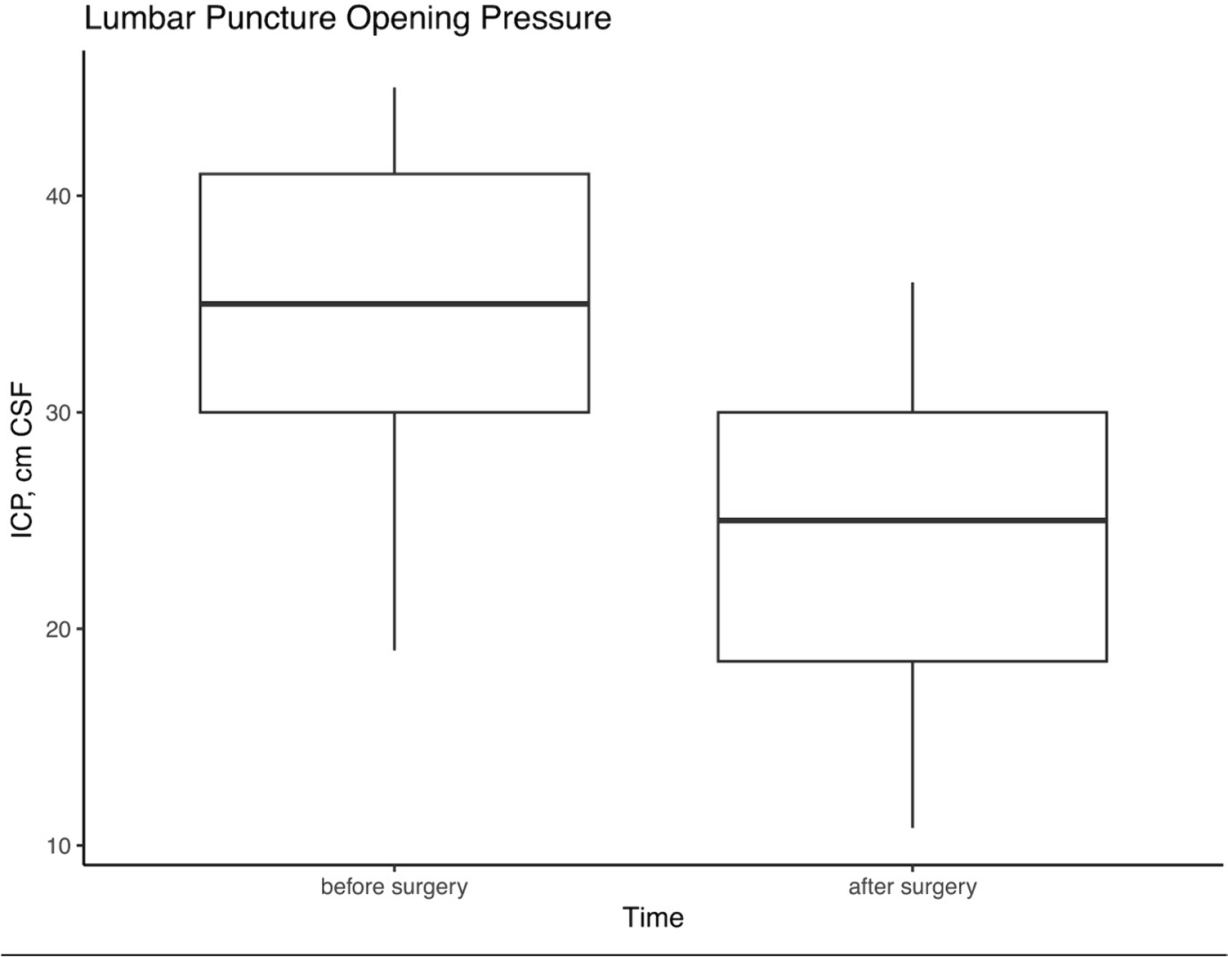
Lumbar puncture opening pressures before and after surgery. Box plot corresponding to 25th, 50th (median), and 75th percentiles

Furthermore, in the subset of 11 patients with available data, intracranial pressure significantly reduced. The mean lumbar opening pressure before and after bariatric surgery was 34.8 ± 8.2 and 24.2 ± 7.6 cm CSF, respectively, with a mean reduction of 10.7 cm CSF (95% confidence interval, 4.7 to 16.6*), p* = 0.003 (Fig. 2). The median number of years elapsed between the preoperative lumbar puncture and the metabolic procedure was 1.4 years (IQR, 0.7–5.1) and that between the metabolic procedure and the postoperative lumbar puncture was 1.68 years (IQR, 0.4–3.1).

A sensitivity analysis was conducted to exclude the 17 patients who underwent invasive procedures for IIH after bariatric surgery. In this subset of 80 patients, there was a significant improvement or resolution in headaches (*p* < 0.001), papilledema (*p* < 0.001), visual field deficits (*p* < 0.001), and visual symptoms (*p* = 0.002). In addition, there was a significant decrease in the number of IIH medications taken postoperatively compared to preoperatively (*p* < 0.001). In 10 patients with available data, the median lumbar opening pressure before and after bariatric surgery was 32.0 (IQR, 29.0–37.0 mmHg) and 23.0 (IQR, 17.5–25.2 mmHg), respectively (*p* = 0.06). The primary findings of study were consistent in the sensitivity analysis.

## Discussion

In this observational study, we found that there was a significant improvement in severity of symptoms and a decrease in the use of medications for patients with IIH after bariatric surgery. At the last follow-up, there was a significant reduction in acetazolamide and topiramate dosages, and also a marked decrease in the number of patients requiring these medications and others for symptomatic control of IIH. Furthermore, the ICP significantly reduced. Our findings suggest that bariatric surgery may be an effective means to control the severity of disease and the reliance on medical treatments.

The basic principles of treatment of IIH are generally aimed at decreasing ICP and preserving vision. First-line management comprises of lifestyle modification and weight loss, followed by medications and invasive treatments as needed [8]. Therapeutic LP can be used during acute IIH flares by quickly reducing ICP to relieve symptoms [9]. However, they are associated with the risk of complications, including meningitis and herniation, and many patients experience recurrence of their baseline symptoms within weeks [10–12]. Similarly, shunting procedures like ventriculoperitoneal shunts function to divert the flow of CSF for more prologued reduction of ICP [13]. However, these can be ineffective in improving symptoms in up to one third of patients, and they incur high risks of serious complications and shunt malfunction that require exchange and removal [5, 14]. Finally, procedures like optic nerve sheath fenestration and venous sheath stenting aim to mitigate the ophthalmologic damage caused by increased ICP. They are effective in reducing papilledema and visual field deficits, but have a high rate of failure and complications, and many patients require subsequent interventions as they are inadequate in controlling other symptoms of IIH [5, 14, 15]. Given that IIH is inextricably linked to obesity, which is in turn linked with diabetes and hypertension, these patients are particularly vulnerable to experiencing complications like infection, wound disruption, and blindness [16].

The mainstay of treatment for IIH is medications. The two medications examined in the present study—acetazolamide, a diuretic, and topiramate, an anti-epileptic—are most commonly used [17]. The therapeutic effects of acetazolamide are thought to be mediated through the reduction of CSF secretion and those of topiramate through its anticonvulsant properties. These medications have been shown to improve papilledema, visual field effects, and ICP. However, they each have their own adverse side effect profiles, like nausea, diarrhea, electrolyte disturbances, cardiac arrhythmias, and metabolic acidosis. Importantly, they do not halt disease progression, and symptom suppression using medication may mask the insidious progression of IIH [18].

Sustained weight loss is essential for long-term remission of IIH and is the first-line treatment option in the absence of fulminant disease that may require more urgent surgical intervention [5]. Weight loss using a 3-month calorie-restricted diet was shown to reduce ICP, papilledema, visual acuity, headache, and use of analgesics [19]. However, it is generally accepted that weight loss by traditional diet control is challenging and difficult to sustain long-term. IIH, which can present with debilitating physical symptoms and is often comorbid with other illnesses like depression, diabetes, and hypertension [20, 21], may further negatively impact adherence to these regimens, making weight loss difficult [22]. Bariatric surgery may be instrumental in helping people with IIH lose and maintain weight, thereby decreasing their IIH symptom burden. In the general population, bariatric surgery has been shown to be superior to lifestyle interventions for weight reduction [23]. Specifically in patients with IIH, it was shown to offer the greatest reduction in body weight, ICP, and headaches, citing the Roux-en-Y gastric bypass as the most effective procedure [24]. Furthermore, Mollan et al. demonstrated that bariatric surgery was superior to community weight management programs for decreasing ICP and increasing quality of life in IIH patients. A follow-up study established that a 24% body weight reduction achieved with bariatric surgery was conducive to a decrease in ICP [25, 26]. Our study contributes to this body of literature by redemonstrating these findings within a longer follow-up time, with a larger study group, and by specifically exploring the dependence on medications and invasive treatments before and after bariatric surgery. The current study compared the extent to which patients relied on medications for IIH before and after bariatric surgery as a proxy for evaluating the severity of disease and found that bariatric surgery had a positive impact. This is a valuable finding for the community of patients with IIH and obesity, who may be counseled on how bariatric surgery fits into the range of treatment options available to them.

There are certain limitations to our study, including those that are inherent to most retrospective analyses. As opposed to those involved in a randomized controlled trial, the patients in our study were selected for bariatric surgery based on their overall obesity-related comorbidities, IIH being only one consideration. While our cohort did not undergo their weight loss operation explicitly to address their IIH, the demographics of these patients and reported preoperative use of medications is generally consistent with the typical group of patients affected by this disease. In our study, patients with IIH were identified with diagnosis codes and confirmed with manual chart review. Preoperative and postoperative symptoms were also confirmed manually, but these symptoms can be nonspecific. For example, headaches and visual disturbances may not be unique to IIH and could potentially have other etiologies in this patient group. However, our results are consistent with the larger body of existing literature on this topic. Furthermore, given the nature of the study, the minimum weight loss threshold which was required for improvement of IIH could not be determined. Future studies should also aim to expand the results over a larger sample size—given the present sample size, we were not able to do multivariable analyses to identify the independent predictors of response to bariatric surgery. Lastly, in our analysis of invasive procedures, we included patients who underwent several shunt and stent revisions but only counted them as one procedure. This may have impacted our results and including them as additional procedures may have been a better representation of symptom burden.

## Conclusion

In this observational study, bariatric surgery among patients with IIH had a favorable sustained outcome with regard to symptom control and reduction of medication use at last follow-up. These results can be used to guide treatment recommendations, especially prioritization of durable weight loss via bariatric surgery, in patients with IIH. Improving access to bariatric surgery for these patients may be accomplished by expanding guidelines for indications for bariatric surgery to include IIH.
